# 8-oxo-7,8-dihydro-2'-deoxyguanosine as a biomarker of oxidative damage in oesophageal cancer patients: lack of association with antioxidant vitamins and polymorphism of *hOGG1 *and *GST*

**DOI:** 10.1186/1756-9966-29-157

**Published:** 2010-12-06

**Authors:** Stéphanie Lagadu, Mathilde Lechevrel, François Sichel, Jean Breton, Didier Pottier, Rémy Couderc, Fathi Moussa, Virginie Prevost

**Affiliations:** 1 Groupe Régional d'Etudes sur le Cancer - UPRES EA 1772 - IFR 146, Université de Caen - Basse-Normandie and Centre de Lutte Contre le Cancer François Baclesse, Caen, France; 2 Laboratoire de biologie moléculaire et cellulaire de la signalisation - EA 3919 - Université de Caen-Basse Normandie, France; 3 CEA Grenoble/INAC/Service de Chimie Inorganique et Biologique, UMR E3 CEA-Université Joseph Fourier, CNRS FRE3200, Laboratoire « Lésions des Acides Nucléiques » Grenoble, France; 4 Service de biochimie, Hôpital Armand Trousseau, AP-HP, France

## Abstract

**Background:**

The present report was designed to investigate the origins of elevated oxidative stress measured in cancer patients in our previous work related to a case-control study (17 cases, 43 controls) on oesophageal cancers. The aim was to characterize the relationship between the levels of 8-oxo-7,8-dihydro-2'-deoxyguanosine (8-oxodG), antioxidant vitamins and genetic susceptibility.

**Methods:**

8-oxodG was analysed in peripheral blood mononuclear cells (PBMCs) by High Performance Liquid Chromatography with Electrochemical Detection (HPLC-ED). Analysis of gene polymorphisms in *GSTM1 *and *GSTT1 *was performed by multiplex PCR and in *GSTP1 *and *hOGG1 *by a PCR-RFLP method. Reversed-phase HPLC with UV detection at 294 nm was used to measure vitamins A and E in serum from the same blood samples.

**Results:**

We observed that in our combined population (cases and control, n = 60), there was no statistically significant correlation between the levels of 8-oxodG and (i) the serum concentration of antioxidant vitamins, vitamin A (*P *= 0.290) or vitamin E (*P *= 0.813), or (ii) the incidence of the *Ser*326*Cys *polymorphic variant (*P *= 0.637) of the *hOGG1 *gene. Also, the levels of 8-oxodG were not significantly associated with polymorphisms in metabolite-detoxifying genes, such as *GST*s, except for the positive correlation with *Val/Val GST P1 *allele (*P *< 0.0001).

**Conclusions:**

The weakness of our cohort size notwithstanding, vitamins levels in serum and genetic polymorphisms in the *hOGG1 *or *GST *genes do not appear to be important modulators of 8-oxodG levels.

## Background

Oesophageal cancer remains an important public health concern worldwide with an estimated burden of 500, 000 new cases in 2005 [1]. The two major histological types of oesophageal cancers, squamous cell carcinoma (SCC) and adenocarcinoma (ADC) differ substantially in their underlying patterns of incidence and key etiologic factors. Alcoholism and smoking are the major established risk factors for SCC, whereas Barrett's oesophagus or gastro-oesophageal reflux disease (GORD) are consistently associated with an increased risk of ADC.

Oxidative stress and reactive oxygen species (ROS) are thought to play a role in oesophageal carcinogenesis. ROS may result from external factors such as smoking, and alcohol metabolism, or may be produced endogenously via inflammatory conditions such as oesophagitis or GORD or may also be due to precancerous lesions (Barrett's oesophagus), as has been shown experimentally in rats [2,3]. Diet influences incidence of oesophageal cancers. An adequate diet of fruits and vegetables is associated with a decreased incidence [4], presumably due to a better supply of antioxidants.

Among the various markers of oxidative stress, 8-oxo-7,8-dihydro-2'-deoxyguanosine (8-oxodG) is particularly popular. It is generated by the oxidation of DNA under physiopathological conditions or environmental stress, but is also a by-product of normal cellular metabolism. It is a premutagenic oxidized-DNA lesion as it is able to mispair with adenine, thus generating G:C to T:A transversion mutations, unless the lesion is repaired prior to DNA replication [5]. Moreover, affordable analytical methods are available for its quantification. In population studies, 8-oxodG is often determined in DNA extracted from white blood cells, peripheral blood mononuclear cells (PBMC) or lymphocytes, although PBMCs being mostly lymphocytes, the distinction is rarely made. These cells are considered to be representative of the whole organism in terms of the level of exposure of to oxidative stress. However, it has been suggested that the apparent high levels of 8-oxodG could be due to artefactual oxidation of DNA during the treatment of the samples. The European Standards Committee on Oxidative DNA Damage (ESCODD) has now been set up within the European laboratory network to improve and harmonise 8-oxodG measurement methods [6-9].

In a previous study [10], we have described the optimisation of an analytical procedure to measure 8-oxodG in PBMCs by using HPLC coupled with electrochemical detection (HPLC-ED). In that study [10], the protocol was applied to the analysis of 8-oxodG in PBMCs of subjects (n = 60) from a case-control study that included both, SCC and ADC cases. Control samples (n = 43) exhibited 4.9 ± 1.9 molecules of 8-oxodG per 10^6 ^unaltered guanosines, levels which correspond to the median values reported by the latest ESCODD trial for HPLC measurement in lymphocytes from healthy young men [11]. In comparison, oesophageal cancer patients (n = 17) showed higher oxidative DNA damage as indicated by the 8-oxodG levels of 7.2 ± 2.6 per 10^6^, 2'-dG (Student's t-test, *P *< 0.001). This difference remained significant even after technical (storage, sampling period, 2'-dG levels) and individual (age, sex, smoking, alcohol) confounding factors were taken into account (*P *< 0.0001, generalized linear regression model). Moreover, data on smoking habits and alcohol consumption of the volunteers were available, and could be correlated with the observed levels of oxidatively-damaged DNA.

The aim of the present study was to characterize the relationship between the levels of oxidative stress, antioxidant vitamins and genetic constitution in oesophageal cancers. An elevated level of oxidative DNA lesions could be related to exogenous or endogenous parameters. Therefore, factors that may influence the extent of oxidative DNA damage such as the nutritional status and genetic polymorphisms were included in this study.

Antioxidant vitamins, such as vitamin A and vitamin E are effective free radical scavengers and can also be useful markers of antioxidant status. Presumably, a higher production of ROS due to severe oxidative stress, characteristic of oesophageal cancers, could lead to a higher metabolic consumption of the antioxidant vitamins, and this would be reflected in their lower serum levels. This "antioxidant hypothesis" was examined in the subjects included in our study by determining the serum concentrations of vitamins A and E.

Oxidatively damaged bases in DNA are preferentially repaired by base excision enzymes. The *hOGG1 *gene encodes the human 8-oxo-guanine DNA glycosylase that cleaves the 8-oxo-guanine base from damaged DNA. The single-nucleotide polymorphism at codon 326 (*Ser*326, rs 1052133) is the most well-studied polymorphism of *hOGG1*. Homozygous carriers of the variant form of the *hOGG1 Ser*326*Cys *gene appear to have a reduced capacity to repair oxidised DNA lesions [12-14], although this is controversial [15]. A genetic susceptibility toward SCC of the oesophagus linked to the *Ser*326*Cys *polymorphism in the *hOGG1 *gene has been described [16]. We have measured this polymorphism in our population and correlated it with the corresponding 8-oxodG level.

Polymorphism also exists in genes encoding enzymes involved in the metabolism of xenobiotics that act as an indirect source of free radicals. Genetic polymorphisms in genes involved in detoxification such as glutathione-S-transferases (GST), *GSTM1*, *GSTT1 *and *GSTP1 *could potentially affect the susceptibility of an individual to the adverse effects of environmental risk factors involved in oesophageal cancer. The above three genes, are expressed in the oesophageal mucosa. We have earlier reported a significant increase in the risk of oesophageal cancers correlated with the null genotypes of *GSTM1 *and *GSTT1 *but not with the *GSTP1 Ile/Val *polymorphism [17]. The polymorphisms in the *GST *genes were analysed according to their histological status, among controls and cases of oesophageal cancers. These polymorphisms were revisited in the present study to investigate their correlation with the levels of 8-oxodG.

## Methods

### Patients and controls

Following an approval from the ethical committee (Comité Consultatif pour la Protection des Personnes en Recherche Biomédicale, Basse-Normandie), consenting patients and control subjects were recruited between 1996 and 2000 within the context of a case-control study aimed at identification of various biomarkers suitable for molecular epidemiology of oesophageal cancers [17]. The control group (n = 43) included healthy donors, who had no clinical history of chronic diseases or cancer and were living in the Lower Normandy, France. Seventeen oesophageal cancer patients from the University Hospital of Caen, France, were selected based on the availability of biological samples. Diagnosis was performed at the Department of Hepato-gastroenterology, University Hospital of Caen, France, and the Department of Anatomopathology, François Baclesse Center, Caen, France. Out of the 17 patients, 9 presented with SCC, 7 with ADC and 1 with leiomyoma, a rare histological subtype. All cases were newly diagnosed and previously untreated. Individual data related to age, sex, alcohol consumption and smoking habits of the subjects have been published earlier [10] and are summarized in Table 1. Twenty ml of venous blood samples were collected before performing any procedure such as surgery, radio- or chemotherapy. The PBMCs were separated and used for quantification of 8-oxodG and genetic polymorphisms from blood samples of all individuals (n = 60), while the serum was used for quantification of the vitamins A and E from all except three samples (n = 57), for which the volumes were insufficient.

**Table 1:** Description of individual data

*Parameter*	*Controls (43)*	*Patients (17)*
**Age (years)**	64 ± 8	63 ± 9
**Sex men/women**	27/16	14/3
**Smoking (cigarettes per week)**	52 ± 79^a^	113 ± 97^b^
**Alcohol (grams/week)**	143 ± 140^a^	289 ± 258^b^

Numbers represent values for mean ± standard deviation; ^a^: n = 36; ^b^: n = 10.

### PBMC collection, DNA isolation and hydrolysis

Care was taken to avoid artefactual oxidation of DNA during its extraction and hydrolysis. PBMCs were isolated from 12 ml out of the 20 ml blood samples using Unisep Maxi tubes (Novamed). These were stored in liquid nitrogen until being used for DNA isolation. Latter was performed using the "protocol G" described by Ravanat *et al*. [18] with modifications aimed at optimisation of the analytical procedure with minimum delays [10]. Other modifications included addition of desferrioxamine to extraction and digestion buffers.

### 8-oxodG HPLC-ED analysis

An optimised method for the quantification of 8-oxodG in PBMCs has been described previously [10]. Briefly, the DNA hydrolysate was analysed by HPLC with an electrochemical detector (Coulochem II; ESA Inc., Chelmsford, MA) using a Supelcosil reversed-phase C18 HPLC column (150 × 3 mm, 5 μm -Supelco) equipped with a C18 guard column. The eluant was 10 mM potassium dihydrogen phosphate, pH 4.6, containing 7.5% methanol, at a flow rate of 0.6 ml/min. The potentials applied to the analytical cell (ESA 5011) were + 50 mV and + 350 mV for E1 and E2, respectively. 2'dG was measured in the same run of corresponding 8-oxodG with a UV detector (Pharmacia LKB VWM 2141) at 290 nm situated after the ED cell. Acquisition and quantitative analyses of chromatograms were carried out using Eurochrom 2000 software (Knauer). The amount of 8-oxodG in DNA was calculated as the number of 8-oxodG molecules/10^6 ^unmodified 2'dG.

### HPLC determination of serum vitamin A and E

Concentrations of vitamins A and E were measured in the sera obtained from the blood samples of all subjects, except for 3 (1 control, 2 patients). The serum fraction was obtained after the isolation of PBMCs from blood by centrifugation at 1000 × *g *for 20 min. Samples from control and cancer subjects were stored in the same conditions, at -80°C for several years until analysis.

Simultaneous determination of vitamin A and E was performed by HPLC as previously described [19], with the following modifications. The HPLC system consisted of a Summit Dual Gradient System including a diode array detector from Dionex (Voisin le Bretonneux, France). The stationary phase consisted of a LiChroCART^® ^125-4 LiChrospher^® ^100 RP-18, 5 μm protected by a guard column filled with the same stationary phase both from Merck Chemicals, France. The mobile phase consisted of methanol and the flow rate was 0.8 ml/min. Separations were carried out at 25°C. Vitamin A and E peaks were integrated at 294 nm and the specificity of the detection was based on retention factors and comparison of UV-Visible spectra with those collected from the standard samples. For the calibration of the method and for quality control we used lyophilised standard and quality control serums from RECIPE (Munich, Germany).100 μL of samples of either serum or a standard solution or quality control sample, were added to 200 μL of a solution of ethanol containing tocopheryl acetate (4 μM) that was used as an internal standard. After stirring the mixture for 30 seconds, the vitamins were extracted with 1000 μL of hexane (2 min of stirring). The organic phase was evaporated under nitrogen and the residues dissolved in 200 μL of methanol and 50 μL were injected into the chromatograph. All procedures were performed in a room with glass windows that prevented penetration of direct sunlight.

### GSTM1, GSTP1, GSTT1 and hOGG1 genotyping analysis

DNA was extracted by the phenol-chloroform method using an aliquot out of the 20 ml venous blood samples of the subjects. Determination of *GSTM1, GSTP1 *and *GSTT1 *polymorphisms in the 60 subjects was performed as previously described [17]. Analysis of deletion polymorphism in *GSTM1 *and *GSTT1 *was performed by multiplex PCR and that of single nucleotide polymorphism in *GSTP1*by a PCR-RFLP method as previously described [20]. In addition to these polymorphisms, subjects were also genotyped for the presence of either the serine or cysteine codon at position 326 (rs 1052133) of the *hOGG1 *gene by PCR-RFLP, using primers and conditions as previously described [21]. Briefly, the PCR amplification of the 293 bp fragment consisted of a 15-min denaturation at 95°C followed by 30 cycles of 95°C for 1 min, 50°C for 1 min and 72°C for 1 min. A final extension step of 72°C for 10 min was included. We used a simple RFLP method to identify the *Ser*326*Cys *by virtue of an *Fnu*4HI restriction site. The *hOGG1 *PCR product was digested with *Fnu*4HI overnight at 37°C. Recovery of two digested fragments (123/124bp and 169/170bp) indicated presence of the *Cys*326 allele, while an undigested amplicon indicated the *Ser*326 allele.

### Statistical analysis

All statistics and graphics have been performed with the SAS System release 9 (SAS Institute Inc., Cary, NC, USA). Distributions of 8-oxodG were normalised by logarithmic transformations. Mean values were compared by Student's t-test or ANOVA and correlations between 8-oxodG and antioxidants were evaluated by Pearson correlation test. All statistical analyses were two-sided.

## Results

### Blood levels of 8-oxodG and vitamins A and E

The mean serum concentrations of vitamin A were 2.77 μM and 2.74 μM, while those for vitamin E were 34.77 μM and 38.73 μM, in patients and controls respectively (Table 2).

**Table 2:** Biochemical parameters of the study group

*Parameter*	*Patients (mean ± s.d.)*	*Controls (mean ± s.d.)*	*P-value^b^* *patient vs. control*
**8-oxodG/10^6 ^2'dG**^c^	7.2 ± 2.6 (*n = 17*)	4.9 ± 1.9 (*n = 43*)	*P *< 0.001
**Vitamin A (μM)**	2.77 ± 0.94 (*n = 15*)^a^	2.74 ± 0.61 (*n = 42*)^a^	*P *= 0.895
**Vitamin E (μM)**	34.77 ± 12.27 (*n = 15*)^a^	38.73 ± 9.47 (*n = 42*)^a^	*P *= 0.204

PBMCs were collected and processed for measuring 8-oxodG. Vitamins were extracted from the serum samples for estimation. ^a ^Numbers in parentheses indicate the number of samples analysed as some samples had to be omitted due to insufficient volume of serum collected. A total of 57 out of the 60 samples were analysed for vitamins. ^b ^Student's t-test was applied. ^c ^Values taken from our data published earlier [10].

**Figure 1:**
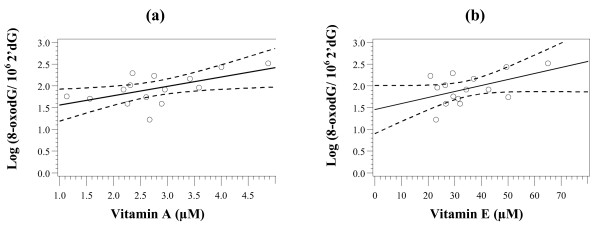
**Correlation between 8-oxodG levels and vitamin A (a) and vitamin E (b) in cancer patients group (n = 15)**. 8-oxodG level is expressed as the number of molecules of 8-oxodG per 10^6 ^2'dG; R = 0.5714 and *P *= 0.026 for correlation between 8-oxodG and vitamin A; and R = 0.4834 and *P *= 0.068 for correlation between 8-oxodG and vitamin E; Log of 8-oxodG (Y-axis) is plotted against vitamin A and E concentrations as indicated; circles, values for individual data; full line, linear regression; dotted line, 95% confidence limit.

There was a high intersample variability in the levels of vitamins across subjects, as indicated by the wide range of values. The mean values in the subjects were in the range of values reported recently by others for these vitamins [22-25]. There were no significant differences in the levels of vitamins A and E between the control and cases. Further, there was no significant correlation found between the levels of 8-oxodG and those of vitamin A (R = 0.1425; *P *= 0.290) or vitamin E (R = 0.0321; *P *= 0.813) when cases and controls were combined (Pearson correlation test, two-sided). However, a positive correlation between the levels of 8-oxodG and vitamin A (R = 0.5714; *P *= 0.026) and vitamin E (R = 0.4834; *P *= 0.068) was observed when only cases (n = 17) were taken into account (Figure 1).

### Levels of 8-oxodG and hOGG1 polymorphism

**Figure 2:**
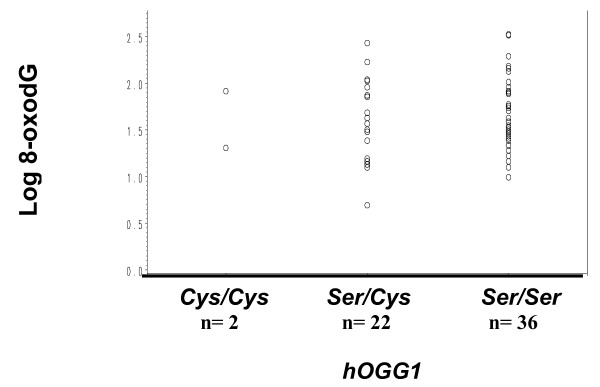
**Levels of 8-oxodG according to *hOGG1 *genotypes**. Data from patients and controls were combined (n = 60) and analyzed by ANOVA (*P = *0.637). 8-oxodG level is expressed as the number of molecules of 8-oxodG per 10^6 ^2'dG and Log of 8-oxodG (Y-axis) is plotted against frequencies of *hOGG1 *genotypes as indicated. circles, values of individual sample.

The potential relationship between 8-oxodG and the *Ser326Cys *polymorphism in the *hOGG1 *gene was examined in the pooled population of cases and controls. Comparisons of means of 8-oxodG between genotypes were done with ANOVA after logarithmic transformation. As shown in Figure 2, there was no statistically significant association between levels of 8-oxodG in DNA and *hOGG1 Ser326Cys *polymorphism (*P *= 0.637). The prevalence of the *Cys *allele, *hOGG1*^326Cys^, was 0.27 in the controls and 0.09 in the cases (Table 3).

**Table 3:** Genotype frequency of hOGG1 Ser326Cys in patients with oesophageal cancer

Genotype	Controls (n = 43) (%)	Patients (n = 17) (%)	Total (n = 60) (%)
***Ser/Ser***	22 (51)	14 (82)	36 (60)
***Ser/Cys***	19 (44)	3 (18)	22 (37)
***Cys/Cys***	2 (5)	0	2 (3)

***Cys *allele frequency**	0.27	0.09	0.22

Numbers in parentheses represent the relative percentage in the group.

### Levels of 8-oxodG and frequency of GST alleles

**Figure 3:**
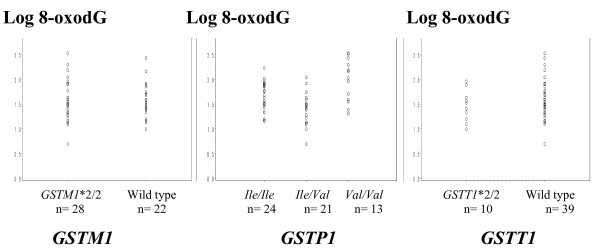
**Levels of 8-oxodG according to genotypes of *GSTM1*, *GSTP1 *and *GSTT1***. Data from patients and controls were combined (n = 60). 8-oxodG level is expressed as the number of molecules of 8-oxodG per 10^6 ^2'dG and Log of 8-oxodG (Y-axis) is plotted against frequencies of the various genotypes as indicated, GSTM1 (P = 0.982), GSTP1 (P < 0.0001 for Val/Val vs Ile/Ile and Ile/Val) and GSTT1 (P = 0.345); circles: values for individual data.

The level of 8-oxodG according to *GST *polymorphisms was examined in the pooled population after logarithmic transformation. There was no difference with the null genotypes of the *GSTM1 *(Student t test; *P *= 0.982), and *GSTT1 *(Student t test; *P *= 0.345), whereas there was a strong difference between GSTP1 variants (ANOVA, *P *< 0.0001) (Figure 3).

## Discussion

Oxidative damage to DNA is considered to be an important risk factor for carcinogenesis. 8-oxodG is a key biomarker in this process because it is one of the most frequently encountered product of oxidatively-damaged DNA and also one that can be easily detected in samples of tissues or urine [26-30]. We have previously reported a significantly higher level of 8-oxodG in circulating blood cells from oesophageal cancer patients compared to control subjects [10]. Similar observations have been made for colorectal carcinoma [31], lung cancer [22,24,32] and leukaemia [33,34]. In our study, none of the individual variables such as smoking, alcohol, sex or age, was shown to influence 8-oxodG concentrations. The aim of the present study was to identify other factors that could modulate 8-oxodG levels. We have attempted to characterize the relationship between oxidative stress, evaluated in terms of levels of 8-oxodG in PBMCs, and the levels of antioxidant vitamins and the genetic constitution, in a population consisting of healthy volunteers and oesophageal cancer patients.

Vitamin C, vitamin E, carotenoids, and other antioxidants present in fruits and vegetables could contribute to cancer prevention by protecting DNA from oxidative damage, according to the "antioxidant hypothesis". By inference, the endogenous levels of these antioxidant vitamins in the serum of oesophageal cancer patients are expected to be low. Likewise, under conditions of severe oxidative stress also, their serum levels may be low as these would be consumed in redox reactions involving ROS.

Many recent epidemiological studies have confirmed that a high intake of fruits and vegetables is associated with a decreased risk of upper aero-digestive tract cancers [4,35-37]. One of the possible mechanisms of this protective effect is the antioxidant activity of vitamins A, C and E. These vitamins are effective antioxidants *in vitro*, and might be expected to protect against cancer. Calişkan-Can *et al*. [24] found lower levels of β-carotene and vitamins A, C and E in lung cancer patients compared to healthy controls. Foksinski *et al*. [23] observed that the mean levels of all the measured antioxidant vitamins were significantly lower in smokers in comparison with non-smokers. Gackowski *et al*. [22] reported that vitamin E levels were significantly reduced in the plasma of lung cancer patients (smokers and ex-smokers) compared to healthy smokers and that the levels of vitamins A and E in plasma of colorectal carcinoma patients were lower than in the control group [31]. In contrast, other studies found no significant differences between healthy smokers and non-smokers for either serum vitamin A or vitamin E [22,38,39]. Also, several large-scale antioxidant supplementation trials have failed to show any clear evidence for a decrease in cancer risk [30,40]. In our study, we found that the endogenous serum levels of vitamins A and E were similar in oesophageal cancer patients and in controls. Notably, despite the fact that our study subjects come from the same geographical area, there was substantial intersample variability, especially for the cancer cases. These differences could reflect the balance between absorption and tissue secretion, and may also be genetically determined. A recording of dietary habits (fruit and vegetable consumption) could have added a complementary and an interesting feature to our study. Determination of the two major water-soluble antioxidants, ascorbate and glutathione would also have brought complementary information. However, as particular conditions are required for sample collection, processing and storage to prevent their oxidation and degradation, these could not be analysed in this retrospective study.

Correlation between the levels of vitamins and 8-oxodG has been reported. In their analyses of 30 cross-sectional studies, Moller & Loft [41] identified 12 studies showing an inverse correlation between oxidatively damaged DNA and antioxidant levels, 16 reporting no correlation and two, a positive correlation. A lack of a correlation between 8-oxodG and antioxidant vitamins has also been reported by others [22,35,42]. In a recent paper, Sram *et al*. [43] found a negative correlation between 8-oxodG and β-carotene and vitamin E but a weak positive association with vitamin A. Similar positive correlations were reported for vitamin A in chemical workers exposed to vinyl chloride monomer [44], carotenoids and vitamin E [45]. We did not find any correlation between the levels of 8-oxodG and vitamins in our study group (cases and controls combined).

Interpretation of these correlative data must be made with extreme caution because the precise effects of antioxidants on mutagenesis and carcinogenesis remain unclear. An antioxidant, including a vitamin antioxidant, is essentially a redox (reduction-oxidation) agent that provides protection against free radicals, but may promote free radical generation under certain circumstances or may exert pro-oxidant effects. Conversely, recent meta-analysis on supplementation trials indicates increased risk of mortality [40], suggesting a pro-oxidant activity at high doses or in cancer-risk subjects (smokers and workers exposed to asbestos). Further, besides vitamins, several food-derived phytochemicals or bioreactive substances in natural products likely contribute to the protection of DNA from damage and thus influence the generation of 8-oxodG *in vivo*. The number of such antioxidants exceeds that of antioxidant vitamins. The availability of these unidentified antioxidants in individual diet could thus affect the correlation between levels of 8-oxodG and antioxidant vitamins. Some dietary components also could up-regulate DNA repair without having any recognised antioxidant function. Interestingly, a positive association was observed in our study between the levels of 8-oxodG and those of the two vitamins, but only in the cases and not in the controls. However, this observation should be interpreted with caution, in the light of the foregoing discussion. Moreover, to arrive at a more convincing conclusion, our data would have to be expanded and adjusted for possible confounders such as age which can become the predominant, independent determinant of oxidative damage as has been discussed recently [43].

In view of the conflicting reports in the literature and the results of the present study, the "antioxidant hypothesis" seems open to criticism. Is there indeed a relationship between antioxidant vitamins and oxidatively-damaged DNA? Secondly, are the concentrations of antioxidants and 8-oxodG in the blood representative measures of the situation in the target tissue of the carcinogenesis and a true reflection of overall cellular DNA damage? Thirdly, do we have reliable tools to examine this correlation? The choice and reliability of biomarkers such as 8-oxodG has also been debated [28,30,46]. The reliability of 8-oxodG is influenced by its method of detection since its artefactual production is a serious concern. Notably, the values of 8-oxodG reported in this study are low and reach the background level of 8-oxodG recommended by ESCODD for HPLC-ED measurement, indicating that these were not an artefact.

It is known that individuals have different responses to oxidative damage and that the risk for oxidative stress-related cancer varies according to both, the environmental exposure and the genetic background. The human 8-oxoguanine DNA glycosylase1 (hOGG1) is one of the major enzymes involved in DNA base excision repair (BER). A positive relationship between *hOGG1 *mRNA expression and 8-oxodG suggests that the expression level of *hOGG1 *may be interpreted as a biomarker of exposure to oxidative DNA damage [47,48]. On the other hand, some studies indicated that there was no interaction between these parameters [12,49,50], which could be explained by the fact that *hOGG1 *is weakly expressed in certain tissues such as the aerodigestive tract tissue [51].

The activity of hOGG1 can be impaired by a polymorphic mutation at codon 326, the *hOGG1 Ser*326*Cys *polymorphism. However, the phenotypic impact of *hOGG1 Ser*326*Cys *polymorphism is unclear. Recent data have shown that the *hOGG1 Ser*326*Cys *polymorphism is associated with a reduced DNA repair capacity for oxidatively-damaged DNA [52], whereas according to others, the converse is true [15,53,54]. This led to the conclusion that both, wild type and the *hOGG1Cys326 *variant-encoded proteins should be functional and probably do not exhibit significant differences in repair activities and hence the polymorphism at codon 326 would probably be neutral [53,55].

Many epidemiological studies have investigated the association of the *Ser*326*Cys *polymorphism in the *hOGG1 *gene indicating an increased risk for head and neck cancers but the reports are conflicting [51,56,57]. Studies on the prevalence of this polymorphism in susceptibility to oesophageal cancer also show conflicting results. Xing *et al*. [16] reported a positive association between the *Cys*326 variant and oesophageal cancer risk in Asians population whereas Tse *et al*. [58] reported no association in Caucasians. In the present study, the small number of samples did not allow us to make a comparison of the genotype distribution between cases and controls in order to determine whether the *hOGG1*^326Cys ^allele contributed to the risk of oesophageal cancer. However, the distribution of *hOGG1 Ser*326*Cys *genotype in our controls (0.44) is in agreement with the frequencies previously described in Caucasian population. This frequency is classically lower than that in Asians [21,51,59], suggesting that this allele may be differently distributed among ethnic groups and may not confer a particular susceptibility to oesophageal cancer in Caucasian population. The allelic distribution of this polymorphism in our combined population followed Hardy-Weinberg equilibrium.

Besides DNA repair activity, enzymes involved in the detoxification of xenobiotics such as glutathione *S*-transferases may influence the extent of oxidative damage in humans. We genotyped our study population for the *GSTM1*, *GSTT1 *and *GSTP1 *genes. Our results indicate no association between *GSTM1 *and *GSTT1 *null polymorphisms and 8-oxodG levels in DNA from PBMCs. On the other hand, we found a statistically significant association between *GSTP1 Val/Val *homozygote carriers and a high level of 8-oxodG (Figure 2). However, as no obvious relationship was found between the frequency of the *Val *allele (*Val/Val *and *Ile/Val *combined) and the level of 8-oxodG, we consider this result questionable. Indeed, correlation of *GST *polymorphisms with 8-oxodG levels in WBCs or lymphocytes varies with the context of exposure: polycyclic aromatic hydrocarbons [60,61], benzene [62], fine particulate matters [63] and hyperbaric oxygen [64].

## Conclusions

In conclusion, although the power of our study is limited, it seems likely that vitamin levels in serum and polymorphisms in the *hOGG1 *or *GST *genes are not important modulators of 8-oxodG levels. Further studies in a larger cohort of patients using other biomarkers of oxidative damage and antioxidant status are required to better understand the possible involvement of oxidative stress in oesophageal cancer.

## List of abbreviations

ADC: adenocarcinoma (ADC); ESCODD: European Standards Committee on Oxidative DNA Damage; GST: glutathione-*S*-transferase; HPLC-ED: HPLC coupled with electrochemical detection; PBMC: peripheral blood mononuclear cells; SCC: squamous cell carcinoma; 8-oxodG: 8-oxo-7,8-dihydro-2'-deoxyguanosine.

## Competing interests

The authors declare that they have no competing interests.

## Authors' contributions

SL: genotyping analysis of polymorphisms, data analysis; ML: interpretation of data concerning polymorphisms, critical revision for important intellectual content; FS: conception and design of the study, interpretation of data, final approval of the version to be published; JB: analysis of 8-oxodG, interpretation of data, critical reading of the manuscript; DP: statistical analysis of the data; RC: interpretation of data concerning vitamins and critical reading of the manuscript; FM: analysis of vitamins and interpretation of these data; VP: coordination of project, interpretation of data and writing of manuscript. All authors have read and approved the final manuscript.
